# A Comparative Analysis of Models for AAV-Mediated Gene Therapy for Inherited Retinal Diseases

**DOI:** 10.3390/cells13201706

**Published:** 2024-10-15

**Authors:** Almaqdad Alsalloum, Ekaterina Gornostal, Natalia Mingaleva, Roman Pavlov, Ekaterina Kuznetsova, Ekaterina Antonova, Aygun Nadzhafova, Daria Kolotova, Vitaly Kadyshev, Olga Mityaeva, Pavel Volchkov

**Affiliations:** 1 Federal Research Center for Innovator and Emerging Biomedical and Pharmaceutical Technologies, 125315 Moscow, Russiavpwwww@gmail.com (P.V.); 2 Moscow Center for Advanced Studies, Kulakova Str. 20, 123592 Moscow, Russia; 3 Institute of Higher Nervous Activity and Neurophysiology, Russian Academy of Sciences, 117485 Moscow, Russia; 4 Research Centre for Medical Genetics, 115478 Moscow, Russia; 5 Department of Fundamental Medicine, Lomonosov Moscow State University, 119992 Moscow, Russia; 6 Moscow Clinical Scientific Center N.A. A.S. Loginov, 111123 Moscow, Russia

**Keywords:** adeno-associated virus, gene therapy, inherited retinal diseases, human retinal organoids, human retinal explants, mouse models, retinal degeneration

## Abstract

Inherited retinal diseases (IRDs) represent a diverse group of genetic disorders leading to progressive degeneration of the retina due to mutations in over 280 genes. This review focuses on the various methodologies for the preclinical characterization and evaluation of adeno-associated virus (AAV)-mediated gene therapy as a potential treatment option for IRDs, particularly focusing on gene therapies targeting mutations, such as those in the *RPE65* and *FAM161A* genes. AAV vectors, such as AAV2 and AAV5, have been utilized to deliver therapeutic genes, showing promise in preserving vision and enhancing photoreceptor function in animal models. Despite their advantages—including high production efficiency, low pathogenicity, and minimal immunogenicity—AAV-mediated therapies face limitations such as immune responses beyond the retina, vector size constraints, and challenges in large-scale manufacturing. This review systematically compares different experimental models used to investigate AAV-mediated therapies, such as mouse models, human retinal explants (HREs), and induced pluripotent stem cell (iPSC)-derived retinal organoids. Mouse models are advantageous for genetic manipulation and detailed investigations of disease mechanisms; however, anatomical differences between mice and humans may limit the translational applicability of results. HREs offer valuable insights into human retinal pathophysiology but face challenges such as tissue degradation and lack of systemic physiological effects. Retinal organoids, on the other hand, provide a robust platform that closely mimics human retinal development, thereby enabling more comprehensive studies on disease mechanisms and therapeutic strategies, including AAV-based interventions. Specific outcomes targeted in these studies include vision preservation and functional improvements of retinas damaged by genetic mutations. This review highlights the strengths and weaknesses of each experimental model and advocates for their combined use in developing targeted gene therapies for IRDs. As research advances, optimizing AAV vector design and delivery methods will be critical for enhancing therapeutic efficacy and improving clinical outcomes for patients with IRDs.

## 1. Introduction

Inherited retinal diseases (IRDs) encompass a diverse array of disorders characterized by the progressive degeneration and dysfunction of various retinal cell types. These conditions result from mutations in over 280 identified genes. Presently, multiple methodologies exist for the investigation and selection of therapeutic interventions for retinal degenerative diseases attributed to genetic mutations. The employment of various models of degenerative diseases facilitates the representation of different stages of disease progression and severity, thereby enhancing the study of pathogenesis and the evaluation of treatment efficacy.

Adeno-associated virus (AAV) vectors have gained substantial traction in the field of in vivo gene therapy related studies, particularly in the context of treating various inherited retinal disorders. These vectors have multiple advantages, such as high production efficiency, low pathogenicity, and minimal immunogenicity [1]. Consequently, AAV-mediated gene therapy presents a promising avenue for addressing autosomal recessive and X-linked disorders restricted to the retina. The use of AAV vectors in gene therapy demonstrates significant potential for the treatment of genetic disorders of the retina. When administered via subretinal or intravitreal injection, these viral vectors exhibit substantial retention within the retina and are largely shielded from neutralization by antibodies. This phenomenon can be attributed to the immune privilege of the retina, characterized by the presence of a blood–ocular barrier, the lack of a lymphatic network within the ocular environment, and localized mechanisms that actively suppress immune responses to introduced antigens [2,3,4]. An example of the successful application of AAV in retinal gene therapy is the FDA-approved treatment of IRDs associated with RPE65 mutations, using AAV2 capsids to deliver a therapeutic cassette containing a functional copy of *hRPE65* (Luxturna, Spark Therapeutics, Philadelphia, PA, USA; 2017, AAV2/2.ssAAV transgene). When administered subretinally, these cassettes were effectively delivered to the retinal pigment epithelium (RPE) at the injection site [5].

A variety of models have been utilized for the proof of concept and preclinical assessment of AAV-based retinal gene therapy, including animal models, human retinal explants, and retinal organoids derived from human induced pluripotent stem cells (hiPSCs). Among these, primates possess ocular structures that closely resemble those of humans; however, their utilization is often constrained by ethical and material considerations. Consequently, experimental ophthalmology frequently employs alternative models, including mice [6], which are favored for their low cost and genetic manipulability; rats [7,8], selected for their similarities in retinal vascularization to that of humans; rabbits [9], which are advantageous due to the size of their eyeballs facilitating surgical procedures; dogs [10], noted for the structural congruence of their retinal layers with those of humans; and pigs, which are utilized because of the similarities in retinal size, structure, and distribution of photoreceptors compared to the human retina [11]. Another advantage of mouse models to be mentioned is the rapid progression of the disease, which, in human subjects, may unfold over the course of several years. Nevertheless, important limitations must be acknowledged, including inherent structural differences in the retinal architecture, a low cone-to-rod photoreceptor ratio, and the absence of the macula [6].

The differences in retinal development, morphology, and structure between animals and humans necessitate the utilization of human-derived models. In addition to animal models, human retinal explants (HREs) can be employed as an ex vivo model for preclinical studies of retinal gene therapies. It is established that there are discrepancies between the tropism and transduction efficacy of different AAV constructs in animal models and human organs. Consequently, the use of HREs in retinal disease models can provide more complete information on the tropism and efficacy of AAV [12]. It is also important to acknowledge the limitations of the HRE model, which precludes its use for the comprehensive development of gene therapies. These limitations include the restricted experimental time frame due to the gradual degradation of the sample, the dependence on the conditions of sample extraction and donor’s medical condition, the inability to study a specific gene of interest, and the lack of assessment of morphological and functional recovery following the transduction of the vector with a healthy copy of the gene [13].

The advancement of methodologies for modeling three-dimensional retinal structures within retinal organoids has significantly improved the investigation of retinal development in vitro, as well as the study of inherited retinal diseases. The major impact in the development of protocols for three-dimensional retinal organoids (ROs) was made with the innovative work of Yoshiki Sasai and his research team, wherein ROs were derived from mouse embryonic stem cells [14]. Currently, established protocols facilitate the generation of ROs from human induced pluripotent stem cells (hiPSCs) and human embryonic stem cells (hESCs), thereby enabling the use of human-derived models in the treatment of inherited retinal diseases and the advancement of personalized medicine. In this context, hiPSCs are obtained from patients with the mutations in retina-associated genes. As a result, hiPSC-derived ROs serve as a highly relevant model for the exploration of gene therapy applications [15]. 

This review aims to conduct a comparative analysis of the principal models used in preclinical gene therapy studies targeting inherited retinal diseases (IRDs). Specifically, it seeks to elucidate the key advantages, limitations, and practical utilities of various experimental models, which include animal models, human retinal explants, and retinal organoids. By detailing the distinct characteristics and challenges present in each type of model system, this review aims to contribute to the broader understanding of how to efficiently model and treat genetic retinal diseases, utilizing the strengths of each model type while acknowledging their respective weaknesses.

## 2. Mouse Models

### 2.1. Pathogenesis of Mouse Models for Retinitis Pigmentosa Type 28 (RP28) and X-Linked Retinoschisis (XLRS)

Mouse models of retinal degeneration serve as invaluable tools in illuminating the pathological mechanisms underlying disease progression, while also facilitating the advancement of therapeutic interventions. Through the deployment of these models, substantial progress has been made in the studying of genetic defects associated with retinal disorders, along with the advancement of gene therapies aimed at reducing disease progression. Furthermore, the genetic homology between mice and humans renders these models particularly suitable for investigating genetic diseases, including inherited retinal diseases such as retinitis pigmentosa (RP) and Leber congenital amaurosis (LCA) [16]. The structural organization of the retina in both murine and human eyes are similar, characterized by several distinct layers [17]. The comparative anatomical framework underscores the potential of murine systems to recapitulate human retinal pathophysiology and expands our understanding of therapeutic approaches in the context of inherited retinal diseases.

In certain instances, the physiological responses of mouse models to experimental interventions diverge from those observed in human subjects [18]. This disparity can be explained by a multitude of factors inherent in the anatomical and functional differences between the two species. Notably, human ocular anatomy is characterized by larger dimensions and a more complex structural composition, including the presence of a macula, which is absent in mice. Furthermore, the proportionally large size of the lens in mouse eyes results in the posterior surface of the lens being in close proximity to the retina, thereby leaving significantly less space for the vitreous, whereas, in the human eye, a larger vitreous chamber is observed. These anatomical variations can significantly influence the outcomes of experimental interventions, requiring caution when extrapolating results from mouse studies to human clinical context. Comparative analysis of retinal morphology reveals that the thickness and structural organization of the mouse retina exhibit remarkable consistency across various regions, including the dentate margin and posterior pole. While the human retina demonstrates significant regional variation, typically exhibiting a thickness approximately twice as great in the peripapillary zone compared to the equatorial region. Differences in gene expression profiles between human and mouse retinal cells may result in different responses to gene therapy interventions. In particular, mouse systems frequently exhibit a heightened immune response to viral vectors utilized in gene therapy applications [19]. Although direct evaluations of gene therapy efficacy in human subjects remain unfeasible, mouse models serve as useful tools for investigating the fundamental biological processes underlying genetic diseases by conducting preclinical studies.

Mice have become representative models of many autosomal recessive and X-linked diseases. One such disease is retinitis pigmentosa type 28 (RP28). RP28 is a recessive retinal disease leading to blindness associated with defects in the *FAM161A* gene. The vast majority of all mutations encountered are located in the large exon 3 of *FAM161A*. Gene therapy targeting *FAM161A* is aimed at restoring its function and preventing retinal degeneration [20]. An analysis of the human retina has demonstrated the localization of FAM161A at the base of the photoreceptor connecting cilia, as well as within the synaptic regions of both the outer and inner plexiform layers, and at the level of ganglion cells [21,22]. Moreover, a number of effective mouse models have been established to study this specific subtype of RP, facilitating further investigations into the underlying mechanisms and potential therapeutic interventions associated with this condition.

A mouse model, *Fam161a*^GT/GT^, was developed to investigate the function of the gene. This model was generated through the introduction of an insertion within exon 3 of the *Fam161a* gene, thereby disrupting the highly conserved UPF0564 domain following amino acid position 362. Quantitative analyses demonstrated that RNA levels in the *Fam161a*^GT/GT^ mice were comparable to those observed in wild-type controls. However, protein level assessments employing Western blotting techniques failed to detect any full-length Fam161a protein in the *Fam161a*^GT/GT^ model. Conversely, immunohistochemical staining methodologies identified the presence of a small quantity of truncated protein. These findings suggest that the *Fam161a*^GT/GT^ model may retain some degree of residual functional activity.

In the context of functional and structural analyses, progressive retinal degeneration in *Fam161a*^GT/GT^ mice manifests at a significantly early stage, prior to one month of age. Longitudinal retinal sections obtained from *Fam161a*^GT/GT^ specimens demonstrate a marked progression of retinal degeneration characterized by a gradual loss of the outer nuclear layer (ONL), which is nearly entirely depleted in mice at five months of age. At three months of age, retinas from the *Fam161a*^GT/GT^ mouse model exhibit an average preservation of only three rows of photoreceptor nuclei; by six months of age, this number diminishes further to a solitary row. Moreover, electroretinography (ERG) assessments indicate abnormal responses as early as one month postnatally, with complete absence of ERG responses recorded by four months of age. Supplemental observations utilizing spectral-domain optical coherence tomography (SD-OCT) imaging corroborate these findings, revealing an early lack of a discernible layer delineating the inner and outer segments of the photoreceptors in *Fam161a*^GT/GT^ mice, thereby providing direct evidence of the progressive nature of retinal degeneration in this model system [20].

In the recently developed *Fam161a* knockout (KO) mouse model, designated as *Fam161a*^tm1b/tm1b^, targeted gene deletion was executed through the removal of exon 3 utilizing a selection cassette engineered with FRT and LoxP sites. Analysis of the resulting model revealed the presence of a truncated RNA transcript encompassing only exons 1 and 2. This transcript is devoid of the crucial functional domains necessary for the protein’s activity, suggesting that the production of a fully functional Fam161a protein is improbable. Furthermore, immunohistochemical assays employing antibodies specifically directed against the truncated isoform of Fam161a, which lacks exon 4, failed to detect the expression of Fam161a in *Fam161a* knockout mice. These findings collectively support the conclusion that the *Fam161a*^tm1b/tm1b^ model does not express functional Fam161a protein.

Based on functional and structural studies, progressive retinal degeneration in *Fam161a*^tm1b/tm1b^ mice begins before one month of age. Notably, the outer nuclear layer exhibits a significant reduction in thickness, with a reduction of more than 90% observed between one month and eight months of age. Histological analyses indicate that the most pronounced and rapid loss of photoreceptor cells in the outer nuclear layer transpires between 1 and 4.5 months of age in *Fam161a*^tm1b/tm1b^ mice. At 8 months of age, only a scant number of photoreceptor nuclei were present, rendering them barely perceptible through functional assessments. It is hypothesized that most of the nuclei that are observed at 8 months of age belong to cells that are no longer functional. In the *Fam161a*^tm1b/tm1b^ mouse model, the complete degeneration of the photoreceptor cell layer becomes apparent around 8 months of age, at which point no discernible electroretinographic response or behavioral reaction to light stimuli is observed. In prior studies involving the *Fam161a*^GT/GT^ model, mice exhibited approximately three rows of photoreceptor nuclei at 3 months of age and reduced to one row at 6 months. Conversely, in the *Fam161a*^tm1b/tm1b^ mouse model, these developmental stages were observed approximately 1 to 2 months later. Optical coherence tomography (OCT) imaging revealed a progressive thinning of ONL in affected specimens over time. Remarkably, by 6 months of age, the ONL was undetectable [10,20].

Degeneration observed in the *Fam161a*^tm1b/tm1b^ mouse model occurs at a slightly slower rate than that seen in the *Fam161a*^GT/GT^ variant. The underlying mechanisms contributing to this discrepancy in degenerative severity remain to be clarified. One potential explanation may involve the detrimental influence of the truncated form of Fam161a on photoreceptor survival, which is expressed in the *Fam161a*^GT/GT^ mouse lineage [23].

Recent investigations into IRDs have raised critical concerns regarding the efficacy of mouse models as substitutes for human pathophysiology. Particularly, mouse models that incorporate human mutant orthologs frequently fail to replicate the exact disease-specific phenotypes observed in human patients [24,25]. This disparity is exemplified in conditions where retinal manifestations can be detected in infants, suggesting that specific lesions may develop before birth. An illustration of this phenomenon is X-linked retinoschisis (XLRS), a disorder precipitated by loss-of-function mutations in the *RS1* gene located on the X chromosome [26,27,28]. To elucidate the mechanisms underlying XLRS, seven distinct mouse models have been developed, including *Rs1*-KO(1), *Rs1*-KO(2), and *Rs1*-KO(3) [29,30,31,32]. Mouse models have demonstrated varying degrees of Retinoschisin (RS1) deficiency and have exhibited characteristic retinal abnormalities, yet they also highlight significant phenotypic divergences [32]. Notably, the median amino acid sequence identity between human and murine *RS1* stands at a relatively low 78.5%, potentially contributing to the differences in disease manifestation [28,33].

The *Rs1*-KO(1) mouse model was produced through the introduction of a lacZ reporter gene in-frame with exon 3 of the *Rs1h* gene, the murine counterpart of the human *RS1* gene, coupled with a neomycin-resistance gene expression cassette [29]. This model has been useful in uncovering remarkable retinal pathology, including disrupted cell layer architecture, compromised integrity of the outer plexiform layer (OPL) and inner nuclear layer (INL), and significant impairments in bipolar cell and rod-associated pathways, as evidenced by electroretinography (ERG). Furthermore, this model has demonstrated atypical cellular displacement within the retinal layers [29,32]. Building on these findings, Zeng et al. [30] established the *Rs1*-KO(2) mouse model by replacing the coding region of exon 1 and a non-coding stretch of 1.6 kb from intron 1 of the *Rs1* gene with a neomycin resistance expression cassette. The *Rs1*-KO(2) model exhibited overlapping retinal abnormalities with *Rs1*-KO(1), particularly in the irregular formation of neuronal and plexiform layers, as well as mislocalization of photoreceptor cells [29,30,32,34].

Meanwhile, the more recent *Rs1*-KO(3) model, established by Liu et al. [31], involved the complete replacement of exons 1 through 3 with a lacZ reporter gene fused in-frame to the *Rs1* start codon. This model revealed a significant reduction in outer retinal thickness by postnatal day 15 (P15) and a rapid onset of photoreceptor cell death by P24 [31,32]. Importantly, both *Rs1*-KO(2) and *Rs1*-KO(3) models demonstrated disturbances in rod transducin and cone arrestin translocation, underscoring visual function deficits prevalent in these experimental setups. Furthermore, the *Rs1*-KO(2) retina displayed heightened susceptibility to light-induced damage even under moderate illumination conditions, highlighting the critical role of genetic background and environmental factors in the pathogenesis of IRDs [30,31,32]. Taken together, these results highlight the limitations inherent in using mouse models as a platform for research into human retinal diseases and therapeutic applications. Although mouse models provide valuable insights into the biological basis of IRDs, the observed phenotypic variations require careful interpretation of the results and highlight the need for additional approaches, including the development of alternative models that can more accurately reflect the complexity of human diseases.

### 2.2. Advancements in Therapeutic Approaches for Retinal Degenerative Diseases in Mouse Models

The pharmacological management of therapeutic interventions encompasses various administration routes, including systemic delivery, topical application, and injection. However, in cases of retinal degenerative diseases, these conventional methods frequently demonstrate limited efficacy due to the presence of numerous anatomical and physiological barriers that impede drug delivery from both the anterior and posterior segments of the eye. Specifically, barriers such as the tear film barrier, blood–aqueous barrier, corneal barrier, vitreous barrier, and blood–retinal barrier significantly restrict the penetration of pharmacological agents into ocular tissues [35]. Furthermore, traditional therapeutic strategies often exhibit reduced applicability in the context of inherited retinal diseases. Consequently, there exists a critical need to develop innovative therapeutic approaches capable of effectively addressing retinal disorders.

Cell-based therapy represents a promising strategy for the treatment of retinopathies, wherein dysfunctional retinal cells are supplanted by newly generated retinal cells in an effort to restore visual function. The degeneration of photoreceptor cells and RPE cells is a hallmark of various ocular pathologies, including age-related macular degeneration (AMD) and retinitis pigmentosa (RP). The loss of photoreceptors, which may occur as a primary pathological event in RP or as a secondary consequence of retinal pigment epithelial (RPE) cell loss in AMD, ultimately culminates in visual impairment or blindness [36,37]. For transplanted retinal cells to achieve a therapeutic effect, it is imperative that they integrate into the host retina, maintain long-term viability, and establish new synaptic connections with existing retinal neurons [38]. Most transplantation studies have employed young post-mitotic photoreceptors or retinal sheets derived from either murine or human retinas (primary cells), as well as photoreceptors generated from pluripotent stem cells (PSCs) of both mouse and human origin. In contrast, the replacement of RGCs presents a more intricate challenge. Given that RGCs extend neural projections, it is essential for transplanted cells to integrate within a complex synaptic network. Furthermore, RGCs must elongate their axons and re-establish functional connections with their specific targets in the central nervous system. Several investigations have demonstrated that both murine and human embryonic stem cells (hESCs) can differentiate into cells exhibiting characteristics of RGCs [39]. 

Gene therapy represents a sophisticated technological approach aimed at achieving therapeutic outcomes by the modification of gene expression in living cells. Such modifications can be performed through ex vivo techniques, wherein cells are altered outside the patient’s body prior to reintegration, or via in vivo methods, which involve direct alteration within the organism [40]. This process typically encompasses the introduction of a functional copy of a defective gene, thereby aiming to restore its physiological function. Currently, viral vectors play a pivotal role in clinical applications for the transduction of target cells. For gene therapy to yield long-term therapeutic benefits, it is imperative to sustain elevated transgene expression levels while concurrently minimizing immunogenic responses. Ideally, vector systems should not integrate into the host genome, exhibit cellular specificity, and maintain a non-toxic profile. Among the various methodologies, recombinant replication-deficient viral vectors—such as adenovirus, lentivirus, and AAV—have been extensively utilized as delivery systems to facilitate the introduction of normal genes into affected retinal tissues, thereby augmenting the expression of functional proteins within target cells [31,41,42,43]. AAV has emerged as a particularly promising vector for clinical applications. The first successful demonstration of the therapeutic potential of gene therapy was obtained in a mouse model of autosomal recessive RP, characterized by homozygous nonsense mutations in the *PDE-β* gene. The protein encoded by *PDE-β* is cGMP phosphodiesterase (PDE), which is crucial for phototransduction. Following intravitreal administration of AAV carrying the *PDE-β* transgene under control of the murine opsin promoter, there was a remarkable increase in photoreceptor amount and improved light sensitivity, confirmed by ERG [44]. This finding underscores the efficacy of gene therapy in addressing the molecular underpinnings of IRDs.

### 2.3. Advancements in AAV-Mediated Gene Therapy for Inherited Retinal Diseases in Mouse Models

Adeno-associated virus (AAV) has been studied as a potential delivery agent in the treatment of IRDs. A viral approach to gene delivery for inherited retinal diseases has presented a safe profile, including minimizing the immune response and reducing integration into the host genome. Bioengineering of AAV capsids have empowered the ability of transduction of a wide range of host cell types. The AAV-based gene therapy is considered to have beneficial potential for inherited retinal diseases, as the most common diseases are those with mutations in the photoreceptors and RPE [4,45]. Since most IRDs are associated with autosomal recessive types of mutations and due to the intricate structure of the retina, it is preferable to maintain the basic visual system by delivering a normal copy of the deficient protein coding sequence (cDNA) into the target of the retinal layers as a gene therapy approach. 

The AAV transduction of the retina has variable tropism on the retinal cell population [46]. Many studies have shown that AAV2/2 is the cornerstone for primary ocular gene therapy research, demonstrating high affinity for retinal ganglion cell (RGC) transduction [47,48]. However, the administration route is considered to be of the most significance in the efficiency of vector penetration [49]. Intravitreal injection of AAV2/2 into the eyes of adult rodents revealed high RGCs transduction efficiency, while most serotypes indicated low transgene expression using the same route of administration [47,48]. Furthermore, the restricted transduction of AAV2/2 to other retinal cell populations in later studies demonstrated the flaw of AAV2/2 as an optimal vector for gene delivery [50,51].

Recently, AAV2/8 has been a commonly used vector for retinal gene therapy, especially in mouse models. In their study, Ferla et al. [52] overcame the packaging capacity limitation of AAV vectors for delivering the large myosin VIIA gene (*MYO7A*) in a knockout mouse model, which is associated with Usher syndrome type 1B (USH1B), by developing dual AAV8 vectors that could encode human *MYO7A*. Dual AAV8.MYO7A was generated from an equal ratio of AAV8 vectors, the first containing the 5′ half of the human *MYO7A* coding sequence and the second containing the 3′ half of the *MYO7A* coding sequence. Different doses of AAV8.MYO7A vectors were administered subretinally into the mouse model. Notably, in the low dose group, the results were moderate and improved over time, while the highest doses of dual AAV8.MYO7A significantly improved retinal structural and functional defects, compared to knockout mice treated with AAV vectors lacking *MYO7A* at the same high dose. Furthermore, the authors suggested that their findings in the treated knockout mouse model proposed a similar type of treatment could be effective in humans at low to moderate doses of dual AAV.

In further studies, AAV8 was also used in subretinal injection procedures to demonstrate that dose-dependent subretinal injection of AAV8 elicited pro-inflammatory peripheral responses to the transgene product. The researchers hypothesized that co-injection of AAV and transgene product peptides may result in a reduction in the anti-transgene immune response to control the immune response associated with subretinal administration of AAV [53].

AAV5, another AAV serotype that has been used in subretinal injection approaches, has been shown to be safe and effective in gene delivery via subretinal administration, particularly in photoreceptor cells and RPE [54,55,56]. McNamee et al. [56] studied the modifier *NR2E3* gene, which is thought to be involved in several pathways including cell survival, oxidative stress, metabolism, apoptosis, and even phototransduction, as a means to reduce disease progression by restoring the homeostasis pathways [57]. Furthermore, a previous study showed that *NR2E3* gene therapy applied to the *Rho^P23H−/−^* mouse model could rescue retinal degeneration associated with RP [55]. The authors examined three different doses of AAV5 containing the *NR2E3* cDNA in a heterozygous *Rho^P23H^* mouse model. They observed the efficacy of the AAV5-hNR2E3 vector doses for up to 6 months after the subretinal injection procedure. The improved photoreceptor cell function and viability in the AAV5-hNR2E3-treated *Rho^P23H+/−^* mouse model were also discovered. Moreover, varying doses of the administered vector reduced retinal degeneration in the *Rho^P23H+/−^* mouse model, and the overexpression of *NR2E3* restored homeostatic pathways in this model by enhancing photoreceptor function and survival, specifically through targeting of the endoplasmic reticulum stress pathway [55,56].

Further studies focused on the efficacy of novel bioengineered AAV vectors in mouse models for gene therapy development. Westhouse et al. [58] performed subretinal and intraretinal injections of various novel AAV vectors. They revealed that AAV2.7m8 was one of the most efficient at transducing via intravitreal route in mouse retina, while other synthetic AAVs tested were not very efficient at transducing mouse retina, such as AAV2-L2 and AAV2-M4. Bioengineered AAV2-M1 and AAV2-1.3 vectors were introduced into mouse retinas showing moderate efficiency. Another study compared two novel engineered capsids (AAV2.GL and AAV2.NN) and AAV2.7m8 in a mouse retina model administered via the intravitreal route. According to their experiments, the researchers demonstrated that eGFP expression delivered by the engineered capsids AAV2.GL and AAV2.NN was higher in the mouse retina compared to AAV2.7m8 administered at the same doses. Moreover, the synthetic AAV2.NN showed the highest tropism in targeting photoreceptor cells [59]. 

Although AAV gene therapy holds great promise, challenges remain in optimizing vector design, delivery methods, and scalability for clinical translation. Importantly, the use of different AAV serotypes has been explored to optimize tissue targeting and transduction efficiency. Thus, variability in serotype efficacy, immune responses to repeated dosing, and the potential for insertional mutagenesis are critical factors to consider.

### 2.4. Analysis of AAV Vector Delivery Methods for Retinal Gene Therapy in Mouse Models

**Figure 1:**
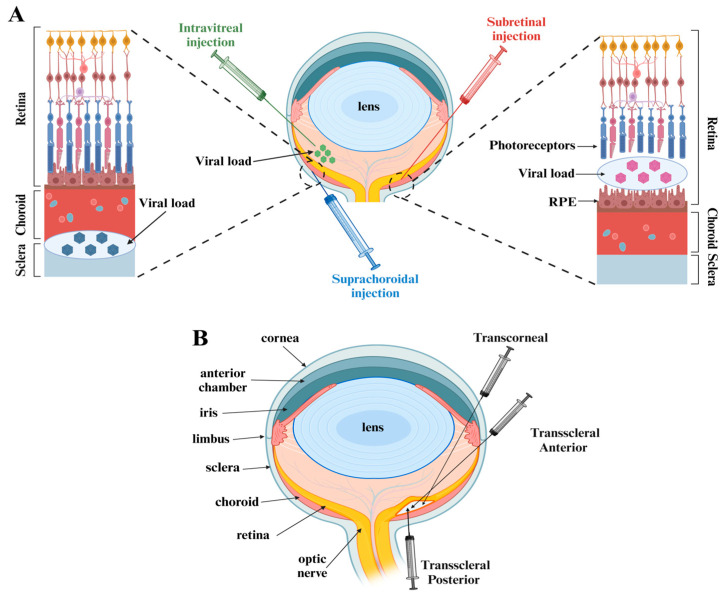
Schematic representation of AAV vector delivery methodologies for retinal gene therapy in mouse models. (**A**) Different routes of administration for AAV vector delivery are illustrated, including intravitreal, subretinal, and suprachoroidal injections. In intravitreal injections, viral delivery is achieved through the vitreous body, with vectors subsequently reaching to the retina. In a subretinal injection, the vectors are delivered to the space between the photoreceptors and the RPE. In a suprachoroidal administration, the AAV are delivered to the area between the sclera and the choroid (**B**) Various subretinal injection techniques are depicted, encompassing transcorneal, posterior, and anterior transscleral subretinal injections. In the transcorneal technique, the needle is inserted through the nasal cornea near the limbus with lens displacement. The transscleral posterior approach is the way through the choroid, while transscleral anterior—the way through the sclerotomy in the limbus. Created with BioRender.com (accessed on 15 October 2024).

Efficient delivery of AAV vectors into the mouse retina is critical for successful transgene expression and therapeutic efficacy. Several delivery routes have been explored for introducing AAV vectors into the mouse retina, each with its own advantages and disadvantages (Figure 1A).

Intravitreal injection (IVT) of AAV into the mouse retina is a method commonly used in gene therapy research to study retinal diseases and develop potential therapeutic strategies. Thus, the eye is typically accessed via the pars plana, employing binocular or microscope assistance for precision. A small amount of AAV vector solution is injected intravitreally using a microinjector and fine needles to ensure accuracy and minimize trauma. After the injection, mice are monitored for any adverse effects such as inflammation or changes in vision. Mice are often observed over several weeks to evaluate the expression of the transgene and any potential therapeutic effects [60]. 

Intravitreal injections are a primary method of vector delivery to target cells, and, in certain cases, they may be the sole option available. X-linked juvenile retinoschisis, a condition that can lead to the splitting of retinal layers or even retinal detachment in advanced stages, necessitates a careful selection of delivery methods [61,62]. In this context, intravitreal injection is preferred over subretinal injection due to the reduced risks of retinal detachment and vitreous hemorrhage associated with the latter approach. While intravitreal injections are typically more effective for treating diseases that impact the inner retina, retinoschisis exemplifies the importance of considering both the type of target cells, the disease’s progression, and the underlying molecular mechanisms when determining the appropriate method of vector administration [63].

Several factors can influence the efficacy of AAV vectors in therapeutic applications. The literature frequently highlights the effectiveness and safety of intravitreal administration of viral vectors in the retina; however, challenges remain due to the dense and viscous nature of the anterior vitreous, which can dilute the vectors as they enter the eye [64,65]. Consequently, the anticipated effects of concentrated transduced vectors may be compromised by the intrinsic properties of the vitreous chamber. The physical characteristics of the vitreous humor continue to pose obstacles to vector efficacy, despite the rational design of AAV capsids that facilitate binding to various cell surface receptors for host cell entry. Various viscous components of the vitreous, such as collagen fibrils, can arrest viral particles, resulting in irregular vector distribution and, consequently, low transduction efficiency [66,67,68].

Recent research has indicated advancements in techniques for intravitreal administration, despite the inherent challenges involved in applying these methods in murine models. Strategies such as the removal of the inner limiting membrane and enzymatic digestion of the vitreous have been explored to optimize intravitreal injections in larger animal models; however, these methods have occasionally resulted in retinal damage. Moreover, optimizing the manipulation of the injection needle could enhance vector delivery by positioning the vectors as close to the retina as possible, thereby facilitating broader distribution throughout the fundus and promoting penetration into the deeper retinal layers [65,67,68].

Subretinal injections are a technique that facilitate localized gene expression while minimizing systemic effects, thereby highlighting its potential utility in therapeutic strategies for inherited retinal diseases. Despite this promise, more investigations are necessary to address challenges related to reinforcing the safety of the procedure and mitigating immune response. While there are some similarities in ocular exposure between subretinal and intravitreal injections, subretinal injections require specific procedural nuances. This technique involves making a small incision in the conjunctiva using a fine surgical blade to create a conjunctival flap. Careful attention is required to avoid damage to the underlying retinal structure, and scleral penetration is typically achieved approximately 1 mm posterior to the limbus. A glass micropipette is then employed to deliver 1–2 µL of AAV solution into the subretinal space. The tip of the micropipette is meticulously inserted into the scleral entry point, with the solution being injected slowly while observing for the formation of a small bleb, which indicates successful localization within the subretinal space. The subretinal injection procedure demands a high degree of accuracy in the delivery of the vector to ensure that loss of vectors into the vitreous does not occur [62,69].

In instances where gene therapy targets the outer layers of the retina, the employment of subretinal injection is strongly advocated. This recommendation is primarily due to the propensity of intravitreal injection techniques to elicit robust inflammatory responses. Subretinal injections mitigate adaptive immune responses, as the administered AAV vectors can achieve localized retention at high concentrations within the subretinal space, resulting in the elimination of transduced cells. Additionally, subretinal injection may facilitate the potential for repeat transduction utilizing AAV particles, owing to the absorption characteristics of the vector suspension into the extracellular matrix of the outer retinal layers [62,70]. Despite the numerous advantages of subretinal delivery, it is important to highlight one of the major limitations—the distribution of administered vectors is commonly localized around the injection site. In this context, a precise locus for the injection must be considered, depending on the specific clinical state of the patient.

It is acknowledged that several methodologies exist for implementing the subretinal injection technique. The age of murine subjects can significantly influence the execution of this procedure; thus, the transcorneal subretinal approach is applicable to adult mice and has demonstrated both efficacy and safety in the postoperative period following in vitro AAV vector administration. However, this technique carries certain risks, including the potential for corneal and lens opacity, multilocal hemorrhages, and damage to photoreceptor segments [62,71].

To circumvent the potential complications associated with the transcorneal approach, the posterior and anterior transscleral approach present a safer alternative (Figure 1B). Both posterior and anterior transscleral methods have been shown to enhance the likelihood of successful transduction while minimizing procedural complications [62]. These techniques have been validated as safe and effective for delivering viral vectors to the subretinal space across various animal models. Notably, approximately fifty percent of mice subjected to subretinal injection experience treatment failure, underscoring a significant challenge in the application of this technique. Nonetheless, subretinal injection continues to be regarded as effective and safe for adult mice when performed with high skill. It should be noted that neonatal mice are preferred candidates for injection via the transscleral route, as this approach mitigates biases introduced by the immature status of ocular structures [62,72].

Suprachoroidal injections, a novel route of administration, have gained prominence for delivering AAV vectors directly into the choroidal space of the eye, facilitating targeted gene expression while minimizing systemic exposure [73]. Traditionally, gene delivery to the retinal tissues has been impeded by anatomical barriers and the intricate structure of the eye. Suprachoroidal administration circumvents some of these limitations by leveraging the interface between the choroid and the sclera, allowing for localized distribution of the vector within the target tissues, including RPE and photoreceptor cells [73,74,75]. This technique has demonstrated promise in preclinical studies, as evidenced by improved transduction efficiency and spatial targeting compared to conventional intravitreal or subretinal injections [76]. Injection of AAV into the suprachoroidal space may result in the viral particles entering the choroidal vessels and the systemic bloodstream due to the fact that the suprachoroidal space is not limited by the haemato–-retinal barrier. Consequently, the transduction efficiency may be reduced, and transduction of non-target tissues and neutralization of vectors by antibodies is a possibility [73].

In suprachoroidal approaches, various types of needles, including microneedles and catheters, may be utilized to facilitate precise control over the delivery of virus vectors into targeted cells. Retinal diseases often impact multiple tissue types; for instance, choroideremia is a condition that affects both the retina and the choroid through the progression of the disease [73,76]. In such cases, suprachoroidal administration of specific vectors enhances bioavailability across the diseased retina and choroid. Given that the vitreous body is considered a barrier to the successful delivery of vectors into the retinal layers, suprachoroidal injections provide a means to circumvent both the inner limiting membrane and the vitreous, potentially improving therapeutic outcomes [76].

## 3. Primary Human Retinal Model

### 3.1. Human Retinal Architecture and Explant Cultures in Retinal Disease Research

A comprehensive understanding of retinal diseases and their potential therapies necessitates extensive research into the human retina, a complex tissue characterized by three primary cellular layers: (1) the ganglion cell layer (GCL), (2) the inner nuclear layer (INL), and (3) the outer nuclear layer (ONL), all of which house the nuclei of principal retinal cell types [77]. 

The ganglion cell layer (GCL) serves as the innermost layer of the retina and consists of the cell bodies of ganglion cells. This layer is positioned adjacent to the vitreous humor and is critical for transmitting neural signals from the retina to the brain through the axons of ganglion cells, which are encapsulated within the retinal nerve fiber layer (RNFL) and extend via the optic nerve [78]. The INL comprises the cell bodies of a diverse array of cell types, including horizontal cells, bipolar cells, amacrine cells, interplexiform neurons, glial Müller cells, and occasionally, displaced ganglion cells. Horizontal cells are primarily located within the inner fibrous portion of the outer plexiform layer (OPL), whereas amacrine cells reside within the inner plexiform layer (IPL) [79]. The ONL contains the cell bodies of photoreceptors, encompassing both rods and cones. These photoreceptors extend their processes to the outer plexiform layer, where they form synaptic terminals. The outer fibers of cones are relatively short, positioning the cone nuclei in a single layer adjacent to the external limiting membrane (ELM). In contrast, the rod cell bodies are organized in multiple rows, situated more internally relative to the cone cell bodies [80]. Moreover, the retina encompasses two synaptic layers, namely the inner plexiform layer (IPL) and the outer plexiform layer (OPL), which facilitate synaptic connections between the ganglion cells and the various inner and outer cellular layers. The most posterior layer of the retina is the retinal pigment epithelium (RPE), which constitutes a monolayer of pigmented hexagonal cells arranged between the neurosensory retina and Bruch’s membrane [77].

**Figure 2:**
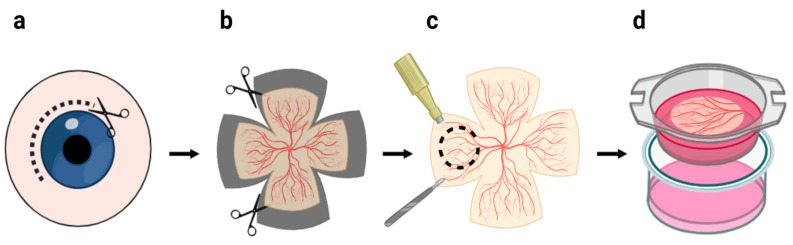
Schematic representation of a streamlined procedure for the harvesting and cultivation of retinal explants [12]. (**a**) A circular incision is made between the transparent cornea and the opaque sclera, after which the anterior segment, lens, and vitreous body are excised. (**b**) The remaining eyecup is subsequently sectioned into four interconnected segments, resulting in a flower-like configuration with four distinct petals. (**c**) Retinal explants are then designated using a round punch, facilitating their separation from the retinal pigment epithelium-choroid complex. (**d**) The retinal explants are transferred into culture inserts within a multi-well plate, where the retinal tissue is cultured for subsequent evaluation in gene therapy studies.

The use of human retinal explant (HRE) cultures as an efficient model for pre-clinical research and gene therapy of retinal diseases is currently a widespread practice, as it preserves the lamination and intercellular composition of the in vivo retina. HREs are commonly derived from ex vivo retinal tissue spanning various developmental stages, ranging from fetal (6.5–8 weeks) to adult, a few hours to a few days after the death of the donor [81]. The quality of the explant is contingent upon the conditions of retinal extraction, including, but not limited to, patients presenting with critical retinal detachment, diabetic eye disease, or vitreoretinopathy [12]. Paramacular regions are considered as the most suitable part of the retina for experimental purposes since they generate reproducible retinal fragments and have a uniform distribution of retinal cell types [82]. The retinal explants are normally derived by dissecting the retina and transferring it to a filter membrane immersed in CO2-independent medium (Figure 2) [83,84].

A study by Orlans et al. [85] demonstrated the effective delivery of gene copies to photoreceptors using AAV vectors in ex vivo human retinal explant cultures. The researchers used AAV2 to introduce a transgene for green fluorescent protein (GFP) to the retinal explants. Initial observations showed no significant expression of GFP for the first 4 days, but by days 6 to 12, there was a notable increase in fluorescence, indicating that about 6 days are necessary for AAV uptake and transgene expression. After a 14-day co-incubation with AAV, immunohistochemical analysis confirmed substantial GFP expression in photoreceptors, particularly in the outer nuclear layer. The researchers also noted concerns about potential retinal tissue degradation from high viral loads over time. 

Beyond the investigation of human retinal explants, the retinal pigment epithelium (RPE), the outermost layer of the retina, warrants particular attention. The primary functions of the RPE include the provision of nutrients to adjacent photoreceptors and the phagocytic removal of shed photoreceptor outer segments, both of which are essential for the maintenance and survival of photoreceptor cells. Consequently, a range of retinal diseases can be traced to dysfunctions originating in or impacting the RPE, leading to the degeneration of not only photoreceptors, but also the underlying choroidal structures [86]. Morphologically, RPE cells exhibit a columnar configuration at the posterior pole, which transitions through a series of alterations in the macular region, characterized by an increase in cell length, a narrowing of cell width, and a heightened pigment density. As the RPE layer approaches the ora serrata, the cells assume a more cuboidal shape and merge with the pigmented epithelium of the ciliary body. This cellular orientation, established during embryonic development, results in the basal aspect of the RPE cells being in close proximity to the choroid, while the apical side interfaces with the neural retina. The basal surface is distinguished by numerous invaginations and forms strong adherence to the underlying basement membrane, which constitutes a part of Bruch’s membrane of the choroid, thereby facilitating a robust attachment to this vascular layer. Despite this intimate association, the RPE is classified within the retinal complex, owing to its derivation from the same embryological germ layer, specifically the neural ectoderm [87].

The retinal pigment epithelium (RPE) represents a pivotal site of pathology in a variety of blinding retinopathies, underscoring the imperative to develop in vitro models of RPE cells for the purpose of investigating these diseases. RPE cultures can be established either from human induced pluripotent stem cells (iPSCs) through directed differentiation or from human postmortem retina specimens. Although both methodologies are deemed acceptable, the establishment of RPE cultures is typically expedited when derived from ex vivo explants. In a study by Engelmann et al. [86], critical principles for the successful cultivation of ex vivo RPE were delineated, namely: (1) the isolation of a sufficient quantity of healthy, viable RPE cells; (2) the stimulation of proliferative activity in RPE cells even following prolonged post-mortem intervals (>48 h) or when sourced from elderly donors; and (3) the prevention of de-differentiation or transdifferentiation, which may manifest as alterations in cellular morphology analogous to fibroblasts, loss of pigmentation, and other phenotypic changes. 

The RPE extraction protocol from ex vivo material involves mechanically isolating the RPE from the eye using tools such as forceps or dissection blades, often in a single sheet or sheet fragments. In general, the RPE extraction protocol is similar to that used for retinal extraction. This method is advantageous as it helps preserve the mosaic-like structure of the RPE. Following cell harvest, the cells are transferred into a culture medium, where they are encouraged to adhere to a substrate that supports the development of a cellular monolayer [88]. The most frequently employed media formulations for cultivating RPE cells include RPMI 1640, MEM, DMEM, and Medium 199. Additionally, using mixed media such as DMEM/F12, supplemented with serum, has become a standard approach for culturing highly differentiated human cells. These conventional culture media offer general culture conditions that are non-selective, promoting the proliferation of various cell types, thereby allowing for the successful establishment of RPE cell cultures [86]. The substrate utilized for the cultivation of RPE cells is generally either the biological matrix Matrigel™ (Corning, New York, NY, USA), or a commercially available polyester transwell membrane. These matrices offer structural support that is akin to the composition of Bruch’s membrane (BrM), thereby facilitating the formation of RPE cells as a monolayer. This configuration plays a critical role in determining the fidelity with which in vitro cultures mimic the characteristics of native RPE cells. It is also important to highlight that RPE cells can be co-cultured with retinal cells in order to enhance retinal viability [89].

### 3.2. Human Retinal Explants for AAV Vector Evaluation

Human retinal explants (HREs) serve as an invaluable ex vivo model system for investigating AAV vectors of various serotypes, facilitating the assessment of their tropism and enabling the identification of the most promising candidates for subsequent clinical trials. The structural characteristics of the AAV capsid and the nuances of the interactions between the viral vector and host cells are widely recognized as critical determinants influencing the efficacy of gene therapy interventions [90]. In addition, the use of retinal explants provides opportunities for directed evolution, thereby enabling the generation of novel AAV variants with enhanced properties [91]. Hence, HREs constitute a convenient and promising model system for advancing research in the domain of gene therapy targeting hereditary retinal diseases.

The predictive validity of gene therapies developed in animal models remains a significant challenge, primarily due to cross-species differences. Consequently, the evaluation of new AAVs in human tissue, prior to clinical application, becomes imperative. Such interspecies discrepancies can profoundly affect critical characteristics of viral transduction [92].

Xi et al. [93] utilized HREs to characterize modified AAV vectors via their previously established methodology termed “scAAVengr”, which uses single-cell RNA sequencing techniques. This innovative approach enabled the identification of three highly effective candidates, canine-derived direct evolution variants (K91 and K912) and AAV2.7m8, that exhibited robust transgene expression within retinal cells [93,94]. Further exploration of capsid development, utilizing HREs as an initial modeling system, was conducted by Westhaus et al. [58]. The authors aimed to evaluate the utility of HREs in the characterization of both natural and synthetic AAV vectors, comparing their performance against other commonly employed model systems, such as hiPSC-derived retinal organoids and RPE cultures. From a comprehensive screening of over 50 AAV capsids, comprising 30 previously reported variants and 21 novel synthetic variants generated through directed evolution, the most promising candidates were identified. Notably, AAV2.7m8, along with AAV2-L2 and AAV2-M4, exhibited a marked tropism for human retinal cells [58,95]. Utilizing the scAAVengr pipeline, researchers can identify the AAVs that exhibit the highest efficiency and broadest transduction across diverse cell types, as well as those that demonstrate specific targeting of particular cell types or desired levels of transgene expression. This information is critical for optimizing gene therapy strategies [94].

Pavlou et al. [59] conducted a study that evaluated the transduction capabilities of AAV2.GL and AAV2.NN in different models, determining human retinal explants to be a highly informative model for the transduction of human cells. Their immunohistochemical analysis, performed ten days post-infection with AAV2.GL and AAV2.NN, revealed significant expression of GFP as a reporter gene in the photoreceptor cells of human retinas. The authors noted the absence of significant in vivo barriers in human retinal explants, such as the internal limiting membrane (ILM), which may influence the variability of viral transduction in human retinal tissues. This research provides a comparative analysis of novel rAAV vectors, highlighting their effectiveness in transducing photoreceptors following intravitreal injections in murine, canine, and non-human primate (NHP) models, as well as in human retinal explants.

HREs facilitate the direct assessment of human-specific promoter activity [96,97,98]. Given that promoter specificity operates independently of the viral delivery method, the necessity for whole-eye in vivo gene therapy is obviated. Promoter activity exhibits significant variability across different species [13,99]. Since promoters critically influence both the strength and cell-type selectivity of transgene expression, it is imperative to evaluate human-specific promoters to optimize gene therapy strategies and ensure their efficacy in human patients [97,98]. Hulliger et al. [97] conducted a functional evaluation of three enhancer/proximal promoter combinations derived from the human *GRM6* gene. This investigation demonstrated that these constructs facilitated robust and highly specific expression in both rod- and cone-type ON-bipolar cells within the human retina, utilizing the AAV2.7m8 viral capsid [13,97]. In their experimental setup, AAV was introduced to the ganglion cell side of cultured HREs. The effectiveness of the promoter was assessed through quantitative real-time polymerase chain reaction (qRT-PCR) of the *crm6* transcript, alongside immunohistochemical detection of the mCitrine reporter gene.

### 3.3. Challenges and Limitations of Human Retinal Explants

While HREs are an invaluable tool for ophthalmic research, they come with several limitations that can affect the scope and interpretation of the results. The degradation of the ex vivo tissue over time is one of the primary challenges to the relevancy of this model. Retinal cells begin to degenerate, and the architecture of the retinal layers can deteriorate, which limits the applicable period of experimental manipulation [100]. 

The progressive decline in physiological functions within the retina is characterized by the deterioration of photoreceptor activity and synaptic transmission. Notably, the axotomy of retinal ganglion cells (RGCs) and the severing of photoreceptor outer segments during procedures such as enucleation and retinal isolation may evoke stress responses associated with axotomy. These responses can subsequently initiate degenerative processes in RGCs, including Wallerian degeneration and the activation of apoptosis. Furthermore, it has been observed that HREs may exhibit a diminished overall capacity for viral transduction. In a study conducted by Nieuwenhuis et al. [99], retinal explants derived from B6.BOla-*Wld*^S^ mice, which are known for their delayed Wallerian degeneration, demonstrated an enhanced transduction efficiency of RGCs despite the occurrence of axotomy. This observation suggests that cellular uptake of viral vectors in post-mortem tissues may be obstructed by various factors, including potential deficiencies in extracellular glycans, cell surface receptors, endosomal trafficking, and gene expression machinery. As a result, genetic constructs that are evaluated within the context of degenerating systems are not intended for straightforward translational or therapeutic applications.

The next aspect to consider is donor-to-donor variability. In contrast to inbred animal models, humans display considerable heterogeneity both genetically and due to environmental exposures. Explants may also vary depending on the donor’s age, health, cause of death, and post-mortem handling, which can influence experimental reproducibility and results [101]. 

This model has notable limitations due to the absence of systemic influences inherent to an intact biological organism. Specifically, the use of explants, which are isolated from their physiological context, precludes the incorporation of systemic physiological factors that may significantly modulate retinal behavior and responses in vivo. Critical elements such as blood supply, immune responses, and hormonal influences remain inaccessible for examination in this isolated setting. Consequently, this limitation may restrict the generalizability of findings derived from the explant model to in vivo conditions. While retinal explants serve as a valuable platform for assessing AAV tropism, the model’s utility in evaluating the mechanisms underlying the delivery of various AAV serotypes to the retina is inherently constrained. Additionally, the lack of a functional immune environment in explants diminishes the capacity to investigate the immunological repercussions associated with different AAV serotypes ex vivo. Therefore, while the retinal explant model may yield insights into certain aspects of AAV biology, its limitations must be acknowledged when extrapolating data to comprehensive in vivo scenarios [102,103,104].

Furthermore, obtaining fresh, high-quality human retinal tissue is challenging due to dependencies on the availability of cadaveric organs and ethical considerations. There are ethical and regulatory guidelines governing the use of human tissues, including the need for donor consent and adherence to specific protocols, which can limit the availability and use of retinal explants [104]. Thus, while HREs can model certain aspects of retinal diseases, they cannot fully replicate the complex in vivo environment of a progressing disease, particularly for conditions influenced by systemic factors or long-term developments.

## 4. Human Retinal Organoid Models

### 4.1. Innovative Approaches to the Development of Retinal Organoids

The retina comprises multiple layers of neurons that establish intricate circuits to transduce light energy into electrical impulses in order to transmit it to the brain. As a prototypical model of neocortical architecture, the mammalian retina has served as a valuable system for investigating sensory processing and neural circuit formation. A primary impetus driving research in retinal development and stem cell biology is the potential to create a sustainable source of healthy retinal neurons for regenerative cell therapies aimed at addressing retinal degeneration and damage. Significant advancements have been achieved in delineating the genetic networks that govern retinal cell fate decisions and their subsequent differentiation [105,106]. These research endeavors have identified transcriptional regulators and extrinsic factors capable of instructing pluripotent stem cells or retinal progenitor cells to commit to retinal lineages and differentiate into specific retinal cell types. However, despite these advances in understanding the mechanisms underlying retinogenesis, establishing retinal neuronal cultures in vitro continues to be challenging. This difficulty primarily stems from the complexity of interactions among various retinal cell types, coupled with the inadequacy of specific signals necessary to orchestrate the distinct stages of retinal cell differentiation. The lack of a suitable model system for in vitro investigations of retinal development and the differentiation of retinal-derived neurons has prompted a renewed focus on the utilization of retinal progenitor cells and pluripotent stem cells. These cellular sources have demonstrated the potential to generate three-dimensional self-organizing retinal tissues, commonly referred to as retinal organoids (ROs). This approach not only facilitates a more accurate recapitulation of the in vivo retinal developmental environment, but also provides a valuable platform for studying the underlying mechanisms of retinal pathogenesis and the evaluation of potential therapeutic interventions.

Over the course of extensive research, RO technology has evolved significantly, beginning with the pivotal discovery that embryonic stem cells possess the capability to self-organize into optic cups [14,107]. Subsequent to this foundational discovery, the initial protocols have undergone numerous refinements aimed at enhancing the viability of specific retinal cell types, optimizing their maturation stages, and improving overall functionality. ROs exhibit a sophisticated laminar architecture akin to that of the human retina, comprising ten distinct layers, each characterized by the presence of various neural cell types. A critical aspect of ROs is the inclusion of three nuclear layers: the outer nuclear layer (ONL), inner nuclear layer (INL), and ganglion cell layer (GCL), alongside two plexiform layers: the outer plexiform layer (OPL) and inner plexiform layer (IPL). A multitude of studies has corroborated the laminar organization of ROs when compared to the human retina, demonstrating the persistence of all relevant retinal cell types throughout a differentiation timeline extending up to 200 days, and achieving this assessment at a single-cell resolution [108,109,110]. These findings substantiate the model of human retinal development and illuminate the mechanisms through which ROs replicate the embryonic developmental trajectory, ultimately reflecting the intricate structural organization characteristic of the human retina.

During the differentiation process from iPSCs to organoids, several pivotal stages are determined. Initially, iPSCs are seeded to facilitate the formation of embryoid bodies (EBs). Importantly, the selected seeding method, whether utilizing a clumped or single-cell protocol, exerts a substantial influence on the subsequent fate of the cells. The early expression of CDH2 (N-cadherin) during the natural aggregation process signifies the occurrence of epithelial-to-mesenchymal transition, a phenomenon that is considered typical throughout embryonic development and is integral to the formation of the neural crest [111]. Conversely, while forced aggregation results in EBs characterized by a higher homogeneity of ectodermal lineage cells, morphological, histological, and functional comparisons reveal no significant differences between organoids derived from the two seeding protocols. This observation suggests the operation of a compensatory mechanism during the neurosphere stage. At this juncture, EBs are cultured in a neural induction medium (NIM) augmented with various supplements to promote the differentiation of cells into neural retinal progenitors. A comprehensive understanding of signaling pathways involved in embryonic development at this stage is essential for effective manipulation of cell fate as organoids mature [112]. Using SAG, a hedgehog pathway activator, during later stages of differentiation facilitates the emergence of retinal-like cells. Moreover, the temporal activation or inhibition of the TGF-β/BMP signaling pathway significantly influences the specification of cells into the neural lineage. For instance, the application of SB431542, a BMP pathway inhibitor, during the initial week of differentiation has been shown to enhance the subsequent development of photoreceptors. Collectively, the regulation of proneural cell fate is a critical feature of the NIM. The next and essential phase in the development of organoids involves differentiation into various retinal cell types and their subsequent maturation. The incorporation of serum, retinoic acid, Taurine, and N2 or B27 supplements significantly contributes to the generation of functional photoreceptors exhibiting distinct inner and outer segments [113].

Matrigel is a widely used extracellular matrix (ECM) component that encompasses a diverse array of natural ECM proteins, including laminin, collagen IV, and heparan sulfate proteoglycans, among others. However, the application of Matrigel is fraught with challenges, such as batch-to-batch variability, which can significantly influence differentiation processes, as well as concerns regarding its animal origin, particularly in the context of clinical applications. Kuwahara et al. [114] proposed an innovative approach to three-dimensional retinal differentiation, utilizing Bone Morphogenetic Protein 4 (BMP4) in a defined culture medium that obviates the necessity for xenogenic matrix protein mixtures. The authors observed that embryoid bodies (EBs) cultured in a growth factor-free chemically defined medium (gfCDM), devoid of both Matrigel and BMP4, predominantly differentiated into Foxg1^+^ telencephalic neuroepithelium, with minimal inclination toward retinal fate. Given the embryonic proximity of the telencephalon and diencephalon, particularly the optic cup, the authors posited that the incorporation of specific signaling molecules could effectively steer differentiation toward a retinal lineage. In this context, the administration of BMP4 from day 6 to day 24, with a gradual reduction in concentration, yielded optimal results, facilitating the most efficient formation of neuroretinal (NR) tissue. It is noteworthy that these conditions primarily enriched the neuroepithelium for NR while lacking retinal pigment epithelium (RPE) and the requisite junctions between NR and RPE. Consequently, the integration of the GSK3 inhibitor CHIR99021 (a Wnt agonist) alongside the FGFR inhibitor SU5402 for a duration of 6 days, commencing on day 18 (to induce RPE), is deemed essential for the subsequent induction of NR through treatments involving fetal bovine serum (FBS), Taurine, and retinoic acid. This multi-faceted approach ultimately facilitates the development of a two-domain organoid composed of NR and RPE.

### 4.2. Exploring Retinal Organoids in the Study and Treatment of Genetic Retinal Diseases

The advancement of RO technology has facilitated the generation of retinal disease models directly from patient-derived somatic cells through the production of iPSCs and subsequent differentiation into retinal cellular phenotypes. These innovative models serve as valuable tools in the initial phases of preclinical research, enabling the evaluation of potential therapeutic interventions and pharmaceutical compounds. Recent efforts in the field have yielded numerous models of genetic retinal disorders, including enhanced S-cone syndrome, retinitis pigmentosa, Leber congenital amaurosis (LCA), glaucoma, retinoblastoma, and X-linked juvenile retinoschisis. Among these pathologies, those exhibiting a recessive mode of inheritance present a particularly promising opportunity for AAV-mediated gene therapy, wherein the introduction of a functional copy of the affected gene demonstrates the potential to achieve therapeutic efficacy. 

Retinitis pigmentosa (RP) is a progressive retinal degenerative disease characterized by a complex genetic landscape. Over 95 genes have been implicated in its development, with a lack of clear genotype–-phenotype correlations complicating the understanding of disease mechanisms. This genetic heterogeneity manifests in various forms of RP, primarily categorized as autosomal dominant (e.g., *RHO*, *PRPF3*, *RP1*), autosomal recessive (e.g., *RPE65*, *PDE6A*, *PDE6B*, *PDE6G*), and X-linked (e.g., *RP6*, *RP23*, *RP24*, *RP34*, *RP2*, *RPGR*) [115]. Related studies have identified mutations in the *USH2A* gene, encoding the usherin protein, as a significant contributor to autosomal recessive non-syndromic RP. Utilizing ROs derived from patients with *USH2A* mutations, researchers have observed significant developmental abnormalities compared to control organoids. These include reduced neuroepithelial thickness, increased cell death, diminished laminin expression, and impaired RPE and photoreceptor function. Further analysis of these *USH2A*-mutated organoids revealed aberrant RPE morphology, characterized by reduced pigmented foci, increased apoptosis, and diminished expression of crucial RPE markers. Concurrently, the expression of genes associated with cilia and dopaminergic synapses was also significantly reduced. Notably, the observed phenotypes in these organoids closely resemble those observed in RP patients, including atrophic pigment mottling on the fundus, thinning of the neuroepithelium and outer nuclear layers, and diminished amplitude of the a-wave and b-wave in electroretinography (ERG) recordings [115]. These findings provide compelling evidence that *USH2A* mutations directly disrupt RPE function, leading to a cascade of detrimental effects on retinal development and ultimately contributing to the progression of non-syndromic RP. This study underscores the potential of RO models as a valuable tool for dissecting the molecular mechanisms underlying specific forms of RP and for developing targeted therapies.

X-linked Retinitis Pigmentosa (XLRP) exhibits significant phenotypic heterogeneity, with mutations in the *RP2* gene accounting for approximately 15% of all XLRP cases [116]. Studies utilizing *RP2*-knockout models and patient-derived ROs have revealed a substantial reduction in photoreceptor populations, particularly rhodopsin-positive (RHO) cells, alongside a decline in ONL and an increase in cell death [117]. Furthermore, investigations of ROs carrying mutations in the *RPGR* gene, another primary genetic contributor to XLRP, demonstrate aberrant photoreceptor development, shortened cilia, and atypical cell death patterns [118]. These findings highlight the critical role of *RP2* and *RPGR* genes in photoreceptor maintenance and the potential of ROs as a valuable tool for studying XLRP pathogenesis.

LCA is recognized as one of the most severe retinal disorders, characterized by an early onset of clinical symptoms. This condition is frequently associated with mutations in at least 28 distinct genes. Pathogenic variants within the *IMPDH1*, *OTX2*, and *CRX* genes exhibit an autosomal dominant inheritance pattern, whereas mutations in other implicated genes typically manifest in an autosomal recessive manner. The genes associated with LCA are integral to the development and functionality of the retina, and their mutations perturb essential processes, including guanine synthesis, photoreceptor morphogenesis, ciliary transport, phototransduction, and the retinoid cycle in both rods and cones [119].

Particularly, mutations in the *AIPL1* gene are linked to Leber congenital amaurosis type 4 (LCA4), a form of severe retinal dystrophy that presents in early childhood. These mutations disrupt the normal functioning of the AIPL1 protein, resulting in a loss or reduction of its chaperone activity. Consequently, this impairment influences the stability and functionality of phosphodiesterase 6 (PDE6), a critical enzyme in the phototransduction cascade within photoreceptor cells. Research has demonstrated that mutations in the *AIPL1* gene lead to altered expression levels of PDE6 in organoids derived from LCA4 patients. Notably, these organoids retain the structural characteristics of the inner and outer retinal layers, at least through the stage of outer segment formation, as evidenced by immunohistochemical analyses and electron microscopy. The maintenance of retinal architecture in these organoids suggests that while the *AIPL1* mutation significantly disrupts specific protein levels and functions, it does not overtly compromise the overall structural integrity of the retina [120].

### 4.3. Application of Retinal Organoids in AAV-Based Gene Therapy for Inherited Retinal Diseases

Retinal organoids (ROs) represent a promising contribution to preclinical investigations for AAV-based therapies. While animal models of retinal degenerative diseases provide essential insights into disease etiology and facilitate the selection of pharmacological candidates for clinical applications, they often fail to accurately recapitulate the complexities of disease progression. Typically, these models exhibit milder phenotypes and considerable variations in retinal development, cellular composition, and molecular profiles compared to human pathology. This limitation emphasizes the necessity for the inclusion of human-derived models, such as ROs, to develop more precise and effective gene therapy strategies. hiPSC-derived ROs can be generated from patients harboring specific genetic mutations, thereby allowing for the exploration of genotype–-phenotype correlations, when applicable.

Among the AAV candidate variants viable for retinal disease gene therapy, AAV8 (Y733F), AAV2 (quad Y-F), AAV2.7m8, rAAV5, rShH10, and rShH10Y-445F have emerged as the most prominent. Findings from studies utilizing hiPSC-derived ROs and retinal pigment epithelium (RPE) cells corroborate these observations, demonstrating that AAV2.7m8 and rAAV5 exhibit superior transduction capabilities [121,122,123]. Furthermore, the administration of a medium containing AAVs to ROs mimics subretinal injection techniques, since in this case AAVs gain access to outer retinal cells [123]. It is crucial to recognize that the efficacy of transduction is significantly influenced by the composition of the medium, as it directly affects extracellular matrix protein availability. For example, the receptor-mediated endocytosis of rAAV2 necessitates heparan sulfate proteoglycan (HSPG) and Fibroblast Growth Factor Receptors 1 (FGFR1) as coreceptors. If fibroblast growth factor 2 (FGF2) is present in the medium, it binds to FGFR1, thereby impeding the transduction efficacy of AAV2 due to the blockage of this coreceptor [121,123]. Moreover, various factors intrinsic to the RO model warrant consideration when utilizing it for AAV-mediated treatment of retinopathies, as they may inadvertently impact viral tropism. These factors include the differentiation protocol, where the timing and selection of added factors can significantly influence the generation of mature photoreceptor-like cells, retinal layer lamination, and the subtype of ROs produced. Additionally, complications arise from the absence of optic nerve innervation, a reduction of ganglion cells during long-term culture, as well as a lack of immune cellular elements and vascular development. Collectively, these variables necessitate careful scrutiny when employing the RO model for study of therapeutic interventions in retinal diseases [113,121].

Retinitis pigmentosa (RP) and Leber congenital amaurosis (LCA) may stem from mutations in the Crumbs homolog 1 (*CRB1*) gene. A study utilizing a mouse model harboring a *CRB1* gene mutation demonstrated that this model accurately reflects the phenotypic progression of the disease. Notably, the localization of the CRB1 protein exhibits distinct differences between mouse and human retinal models. In murine retinas, CRB1 is primarily localized to Müller glial cells within the subapical region, whereas in human fetal retinas and hiPSC-derived ROs, CRB1 is observed in both photoreceptors and Müller glial cells at the subapical region [124]. Boon et al. [124] advanced their investigation by developing a hiPSC-derived RO model featuring a homozygous mutation in the *CRB1* gene. For the transduction of the ROs, the researchers employed AAV vectors; AAV5.CMV.GFP and AAV2.CMV.GFP. Due to AAV5 possessing higher tropism for Müller glial cells than AAV2, they administered AAV5 on the 120th day of differentiation, specifically for subsequent AAV.hCRB gene augmentation therapy at an intermediate dosage of 3.3 × 10^10^ viral genomes (vgs). Notably, the researchers noted that AAV5 efficiently transduced retinal pigment epithelium (RPE) cells. Upon treatment of the *CRB1*-deficient ROs, analyses conducted on days 180 and 210 of differentiation revealed an increase in outer nuclear layer (ONL) thickness with no significant changes observed in the thickness of the retinal or inner nuclear layer (INL). Additionally, there was an increase in the number of photoreceptor nuclei alongside a decrease in the number of photoreceptor nuclei protruding above the outer limiting membrane, a hallmark of the pathological condition, indicating a partial recovery of the RO phenotype.

One significant etiological factor contributing to severe XLRP is a homozygous mutation in the *RP2* gene. Although knockout mouse models for the *RP2* gene have been established, these models fail to accurately replicate the severe phenotypic manifestations observed in affected human patients. For example, patients manifest macular atrophy in early childhood, while the mouse models exhibit milder phenotypic features that do not become apparent until later stages of development, specifically at 2, 5, or 14 months of age, contingent upon the particular model. In their study, Lane et al. [117] employed an hiPSC-derived RO model characterized by a mutation in the *RP2* gene. Following differentiation of the *RP2*-deficient RO beyond 150 days, a significant reduction in photoreceptor-like cells, predominantly rod photoreceptors, was noted, alongside indications of cellular apoptosis and thinning of the ONL. These observations coincided with the developmental period of rod photoreceptor maturation and rhodopsin expression. Furthermore, the *RP2*-deficient ROs were subjected to treatment on day 140 with AAV5.CAGp.RP2 and subsequently evaluated 40 days post-treatment. This intervention resulted in an observable increase in ONL thickness, with RP2 being detected on the membranes of both rod and cone photoreceptors. To facilitate a comprehensive analysis of the application of ROs in AAV-based gene therapy, Table 1 presents a synthesis of several recent investigations that report outcomes utilizing AAV-mediated gene therapy for the treatment of inherited retinal diseases. These studies encompass a diverse array of ROs associated with a range of genetic mutations relevant to different pathological conditions.

Currently, ROs constitute the most advanced in vitro system for the investigation of the mechanisms underlying inherited retinal diseases. This model also offers a relevant human retinal context for assessing the efficacy of gene expression mediated by viral constructs.

## 5. Conclusions

The exploration of retinal models for AAV-based gene therapy continues to reveal significant insights into the treatment of inherited retinal diseases (IRDs). This comprehensive review highlights the distinct advantages and limitations of various model systems, including in vitro retinal organoids, ex vivo human retinal explants, and in vivo mouse models. Each approach offers unique benefits: mouse models provide a platform for genetic manipulation and disease progression analysis; human retinal explants serve as a bridge between in vitro studies and clinical applications; and ROs facilitate the modeling of human retinal development and disease phenotypes.

The exploration of retinal models for AAV-based gene therapy continues to reveal significant insights into the treatment of inherited retinal diseases (IRDs). This comprehensive review highlights the distinct advantages and limitations of various model systems, including in vitro retinal organoids, ex vivo human retinal explants, and in vivo mouse models. Each approach offers unique benefits: mouse models provide a platform for genetic manipulation and disease progression analysis, which is more appropriate for testing the systemic effects of the drug; human retinal explants serve as a bridge between in vitro studies and clinical applications, an optimal testing ground for vectors, promoters and genetic constructs; and ROs facilitate the modeling of human retinal development and disease phenotypes.

This comparative analysis underscores the vital role of these diverse model systems in advancing our understanding of genetics, pathophysiology, and therapeutic interventions in IRDs. With the promising potential of AAV vectors for delivering therapeutic genes, ongoing research must focus on optimizing vector design, improving delivery methods, and minimizing immunogenic responses. As the field moves toward clinical translation, the integration of these models will be paramount in refining gene therapy strategies tailored for individual patient needs. Ultimately, the continued innovation in retinal model systems offers hope for developing effective treatments and restoring vision for those afflicted with IRDs.

## Figures and Tables

**Table 1:** Summary of patient-derived retinal organoids in inherited retinal diseases.

Disease	Gene	Inheritance Type	Retinal Organoid Differentiation Procedure	Neural Induction Media (NIM)	Retinal Differentiation Media	AAV Type	Transduction-Readout Time Point	Main Results	Reference
RP or LCA	*CRB1*	autosomal recessive disease	EB formation then transferred to adherent culture, NRV isolation and cultivation in suspension. Retinal organoid differentiation was carried out as reported previously with some modifications [125]	D1: mTesR/NIM 3:1 (DMEM/F12 1:1, N2, NEAA, 2 μg/mL heparin) D2: mTesR1/NIM 1:1 D3–D15: 100% NIM	D16–D34: DMEM/F12 3:1, B27, NEAA D35–D6\3: +FBS, 100 µM Taurine D64–D84: +1 µM RA D84–D119: +0.5 µM RA Dd120–end—without RA	AAV5.CMVmin.hCRB1 or AAV5.CMVmin.hCRB2	D120–D180, D210	Increased number of photoreceptor nuclei. Increased CRB1 localization at the OLM after AAV.hCRB1 treatment. Overexpression of CRB2 in photoreceptor cells after AAV.hCRB2 treatment. Increased thickness of the ONL, but not the retina or the INL. Restoration of gene expression related to the endosomal system to isogenic control levels.	[124]
X-linked RP	*RP2*	X-linked recessive disease	EB formation then transferred to adherent culture, NRV isolation and cultivation in suspension. Retinal organoid differentiation was carried out as reported previously with some modifications [125].	D1: E8/NIM (DMEM/F12 1:1, N2, NEAA, 2 μg/mL heparin) 3:1 D2: E8/NIM 1:1 D3–D15: 100% NIM	D16–D41: DMEM/F12 3:1, B27, NEAA D42–D63: +FBS, 100 μM Taurine D63–D91: + 1 μM RA D92–D139: +0.5 μM RA D140–end: without RA	AAV5.CAGp.RP2	D140–D180	ONL thickening close to endogenous control. The percentage of rhodopsin-positive cells was above average in the non transduced RP2 KO. The percentage of cone arrestin-positive cells was reduced after AAV transduction. The rescue of the ONL thinning phenotype in RP2 KO ROs suggests a protective effect of RP2 overexpression in photoreceptor cells.	[117]
X-linked RP	*RPGR*	X-linked recessive disease	Adherent culture, NRV isolation and cultivation in suspension. Retinal organoid differentiation was carried out as previously reported [126,127].	D3–D28-42: DMEM/F12 (1:1), NEAA, N2 before NRV formation	D28–42–D69: DMEM/F12 3:81, FBS, B27, 100 μM Taurine D70–D83: +1 μM retinoic acid D84–D99: +N2, 0.5 μM RA D100–end of differentiation: without B27 and RA	AAV2.7m8.hRKp.PGRORF15	D135–D160	CRX-positive photoreceptor cells within the ONL. The extension of recoverin positive OS into the peripheral space. Significantly upregulated expression of the RPGRORF15 transcript.	[128]
LCA4	*AIPL1*	autosomal recessive disease	Adherent culture, NRV isolation and cultivation in suspension. Retinal organoid differentiation was carried out as previously reported [127,129].	D3–D28-42: DMEM/F12 (1:1), NEAA, N2 before NRV formation	D28–42–D69: DMEM/F12 3:1, FBS, B27, 100 μM Taurine D70–D83: +1 μM RA D84–D99: +N2, 0.5 μM RA D100–end: without B27 and RA	AAV2.7m8.hRKp.AIPL1	D161–D175, D219, D231	Rod OS structures increased significantly in length, and the abnormal accumulation of rhodopsin in the somas of patient rods was abolished. L/M-opsin cone OSs also recovered significantly and CEP290 protein became detectable. Localization of other OS proteins, including visual arrestin, peripherin2 phosphodiesterase 6B, and rod α-transducin, was restored to varying degrees. Reduced levels of S-opsin were mislocalized to axons and synaptic pedicles. OS biogenesis was at least partially rescued.	[130]
LCA and renal-retinal Senior-Løken syndrome	*IQCB1*/*NPHP5*	autosomal recessive disease	EB formation then transferred to adherent culture, NRV isolation and cultivation in suspension. Retinal organoid differentiation was carried out as previously reported, with some modifications [131].	D1: E8/NIM 3:1 (DMEM/F12 1:1, N2, NEAA, 2 μg/mL heparin) D2: E8/NIM 1:1 D3–D15: 100% NIM	D16–D28: DMEM/F12 3:1, B27, NEAA/NIM 3:1 D28–D41: +20 ng/mL IGF-1 D42—D62: +FBS, 100 µM Taurine D63—D90: +1 µM 9-cis-retinaldehyde D91–D119: +0.5 µM 9-cis-retinaldehyde D120–end: replacing FBS with KSR	AAV2.CMV.NPHP5	D120–D150, D200	Upregulation of genes primarily associated with innate immunity or interferon-induced viral responses. Remarkable recovery of PDE6α and PDE6β was observed in both models, indicating that AIPL1 function in the ONL was restored.	[132]
RP	PRPF31	autosomal dominant disease	Adherent culture, NRV isolation and cultivation in suspension. Retinal organoid differentiation was carried out as previously reported [133,134].	D0–D2: TeSR-E6 D3–D27: +N2	D28–D34: DMEM/F-12, 1:1, NEAA, B27, 10 ng/mL FGF2 D35–D83: +FBS, without FGF2 D84–D200: DMEM/F-12, B27, NEAA	AAV2.7m8.CAG.PRPF31	D85–D175	Displayed ~40% NRL-positive rods and 20% hCAR-positive cones. Increasing PRPF31 expression levels directly, preventing photoreceptor degeneration in mature retinal organoids transduced before the first signs of degeneration.	[135]
LCA	CRX	autosomal dominant disease	EB formation then transferred to adherent culture, NRV isolation and cultivation in suspension. Retinal organoid differentiation was carried out as previously reported with modification of culturing dissected retinal organoids individually in a 96-well plate format [131].	D1: E8/NIM 3:1 (DMEM/F12 1:1, N2, NEAA, 2 μg/mL heparin) D2: E8/NIM 1:1 D3–D15: 100% NIM	D16–D28: DMEM/F12 3:1, B27, NEAA/NIM 3:1 D28–D41: +20 ng/mL IGF-1 D42–D62: +FBS, 100 µM Taurine D63–D90: +1 µM 9-cis-retinaldehyde D91–end: +0.5 µM 9-cis-retinaldehyde	AAV2.CRX.CRX	D120–D150, D180	Increased CRX mRNA and protein in treated organoids. Rescued rhodopsin expression. Partially restored L/M opsin expression. Long-term expression of CRX did not result in activation of the apoptotic marker Caspase3.	[136]

Abbreviations: RP, Retinitis pigmentosa; LCA, Leber congenital amaurosis; *CRB1*, Crumbs-homologue-1; *RP2*, Retinitis Pigmentosa 2; *RPGR*, RP GTPase regulator; *AIPL1*, aryl hydrocarbon receptor interacting protein-like 1; *IQCB1/NPHP5*, Q calmodulin-binding motif containing B1/nephrocystin-5; *PRPF31*, pre-mRNA processing factor 31; *CRX*, Cone-rod homeobox; CMV, Cytomegalovirus; CAG, CMV early enhancer/chicken β actin; NRV, neuroretinal vesicle; EB, embryonic bodies; RA, retinoic acid; FBS, fetal bovine serum; KSR, knockout serum replacement, NEAA, MEM non-essential amino acids; FGF2, Fibroblast growth factor 2; IGF-1, Insulin-like growth factor 1; ONL and INL, outer and inner nuclear layer; OS, outer segment of photoreceptor.
